# Use of HLA-B27 tetramers to identify low-frequency antigen-specific T cells in *Chlamydia*-triggered reactive arthritis

**DOI:** 10.1186/ar1221

**Published:** 2004-09-23

**Authors:** Heiner Appel, Wolfgang Kuon, Maren Kuhne, Peihua Wu, Stefanie Kuhlmann, Simon Kollnberger, Andreas Thiel, Paul Bowness, Joachim Sieper

**Affiliations:** 1 Charite Berlin, Campus Benjamin Franklin, Department for Gastroenterology, Infectiology and Rheumatology, Berlin, Germany; 2 Deutsches Rheumaforschungszentrum Berlin, Germany; 3 MRC HIU, Institute of Molecular Medicine, John Radcliffe Hospital, Oxford, UK

**Keywords:** HLA-B27, T cells, tetramers, reactive arthritis

## Abstract

Reports of the use of HLA-B27/peptide tetrameric complexes to study peptide-specific CD8^+ ^T cells in HLA-B27^+^-related diseases are rare. To establish HLA-B27 tetramers we first compared the function of HLA-B27 tetramers with HLA-A2 tetramers by using viral epitopes. HLA-B27 and HLA-A2 tetramers loaded with immunodominant peptides from Epstein–Barr virus were generated with comparable yields and both molecules detected antigen-specific CD8^+ ^T cells. The application of HLA-B27 tetramers in HLA-B27-related diseases was performed with nine recently described *Chlamydia*-derived peptides in synovial fluid and peripheral blood, to examine the CD8^+ ^T cell response against *Chlamydia trachomatis *antigens in nine patients with *Chlamydia*-triggered reactive arthritis (Ct-ReA). Four of six HLA-B27^+ ^Ct-ReA patients had specific synovial T cell binding to at least one HLA-B27/*Chlamydia *peptide tetramer. The HLA-B27/*Chlamydia *peptide 195 tetramer bound to synovial T cells from three of six patients and HLA-B27/*Chlamydia *peptide 133 tetramer to synovial T cells from two patients. However, the frequency of these cells was low (0.02–0.09%). Moreover, we demonstrate two methods to generate HLA-B27-restricted T cell lines. First, HLA-B27 tetramers and magnetic beads were used to sort antigen-specific CD8^+ ^T cells. Second, *Chlamydia*-infected dendritic cells were used to stimulate CD8^+ ^T cells *ex vivo*. Highly pure CD8 T cell lines could be generated *ex vivo *by magnetic sorting by using HLA-B27 tetramers loaded with an EBV peptide. The frequency of *Chlamydia*-specific, HLA-B27 tetramer-binding CD8^+ ^T cells could be increased by stimulating CD8^+ ^T cells *ex vivo *with *Chlamydia*-infected dendritic cells. We conclude that HLA-B27 tetramers are a useful tool for the detection and expansion of HLA-B27-restricted CD8^+ ^T cells. T cells specific for one or more of three *Chlamydia*-derived peptides were found at low frequency in synovial fluid from HLA-B27^+ ^patients with Ct-ReA. These cells can be expanded *ex vivo*, suggesting that they are immunologically functional.

## Introduction

*Chlamydia*-triggered reactive arthritis (Ct-ReA) is strongly associated with HLA-B27 like other spondylarthropathies, and especially ankylosing spondylitis [1,2]. ReA occurs 1 to 4 weeks after urogenital infection with *Chlamydia trachomatis *or gastroenteral infection with enterobacteria such as *Yersinia enterocolitica *[3]. After acute onset, most patients have a self-limiting course, but up to 20% suffer from a disease duration of more than 1 year [4]. Of HLA-B27^+^-reactive arthritis patients, 20–40% move on to ankylosing spondylitis after 10–20 years, suggesting that the ReA-associated bacteria can cause ankylosing spondylitis [5] and that immune mechanisms triggering the disease are induced by T cell responses to microbial antigens. The main hypothesis advanced for the association between HLA-B27 and spondylarthropathies is the arthritogenic peptide theory. It states that some HLA-B27 subtype alleles, owing to their unique amino acid residues, bind a specific arthritogenic peptide that is recognized by CD8^+ ^T cells [6-9]. Recently we and several other groups have reported on *Chlamydia*-specific CD8^+ ^T cells capable of lysing target cells primed with *Chlamydia *antigens [10-12]. CD8^+ ^T cell responses in spondylarthropathies other than Ct-ReA have also been described [13-15].

Recently a new method for antigen-specific T cell recognition has been established by using multimerized MHC/peptide molecules [16]. These molecules are called tetramers because they contain four soluble and biotinylated MHC molecules linked to labelled streptavidin that specifically bind with high avidity to T cell receptors. In comparison with intracellular cytokine staining, the major advantage of tetramer technology is the identification of antigen-specific T cells independently of their cytokine secretion profile, the possibility of sorting unstimulated T cells and of having a tool for the antigen-specific detection of T cells in experiments *in situ *[17].

In humans, MHC class I tetramers are widely used, and HLA-A2 tetramers in particular are an important tool in tumour immunology [18]. However, the use of HLA-B27 tetramers in HLA-B27-related diseases is rare [10,19]. The rarity of their use might be related to heavy protein aggregation during the refolding procedure of the recombinant HLA-B27 monomer [19,20]. To determine optimised conditions for the refolding procedure of soluble HLA-B27 monomers with bacteria-derived epitopes we first used HLA-B27 tetramers with a well-described HLA-B27-restricted viral epitope from Epstein–Barr virus (EBV). We analysed the refolding rate of HLA-B27 monomers and compared our results with refolding gained with an HLA-A2 molecule loaded with a viral epitope from EBV [21]. On the basis of these results we applied the HLA-B27 tetramer technology to specify the HLA-B27-restricted CD8^+ ^T cell response to *Chlamydia*-derived peptides in patients with Ct-ReA.

This is the first report of a systematic use of HLA-B27 tetramers in humans in an HLA-B27-related disease.

## Methods

### Patients

We analysed six HLA-B27^+ ^and three HLA-B27^- ^patients with ReA after infection with *Chlamydia trachomatis *(Table 1). We diagnosed ReA if patients had a prior urogenital infection, which was confirmed by the detection of *Chlamydia trachomatis *in the morning urine by polymerase chain reaction. An additional criterion was the detection of *Chlamydia*-specific antibodies [6] at the beginning of the disease or highest synovial T cell proliferation against *Chlamydia trachomatis *[22] in proliferation assays with whole *Chlamydia *antigen. The results were compared with tetramer staining in six HLA-B27^+ ^healthy blood donors. We also examined synovial T cells from three HLA-B27^+ ^patients with ReA after gastroenteritis and having highest synovial proliferation against enterobacteria. We also tested the synovial fluid of three patients with rheumatoid arthritis. In addition we used HLA-B27^+ ^and HLA-A2^+ ^blood donors with previous EBV infection for experiments comparing HLA-B27 and HLA-A2 tetramers.

The ethical committee of the Benjamin Franklin Medical Centre gave ethical approval for this study.

### Search for peptide binding affinity

The quantification of HLA-B27 binding affinity was conducted with two different programs that analyse HLA-peptide binding motifs, one called SYFPEITHI described by Rammensee and colleagues [23] and the other called BioInformatics and Molecular Analysis Section (BIMAS; ).

### Peptide synthesis

Nonamer peptides were synthesized by standard 9-fluorenyl-methyloxy-carbonyl solid-phase synthesis methods on a Syro-Synthesizer (MultiSyn Tech, Witten, Germany), purified by high-performance liquid chromatography (Shimadzu LC-10; Shimadzu Scientific Instruments, Duisburg, Germany) and identified by mass spectroscopy (LCQ, ion trap; Thermoquest, Eberbach, Germany). The purity of the peptides was more than 95%. Peptides were dissolved in dimethyl sulphoxide. For T cell stimulation and fluorescence-activated cell sorting (FACS) analysis of intracellular cytokine staining, the peptides were further diluted with serum-free medium at a concentration of 5 mg/ml and frozen at -80°C.

### FACS analysis of antigen-specific T cells with HLA-B27 tetramers

HLA-B27 tetramers were generated as described previously [19], with some modifications. The expression vector pLM1-HLA-B27 was modified by tagging with the BirA recognition sequence as described previously and by mutating the cysteine residue at position 67 to serine. After being refolded, the recombinant protein was concentrated and centrifuged at 13,000 rpm (16,060*g*; Haereus Biofuge Pico; Kendro Laboratories, Langenselbold, Germany) followed by biotinylation and gel filtration with a Superose 12 column (Pharmacia) on an Äkta Basic system (Pharmacia). Correct folding and biotinylation were analysed by gel filtration (Äkta Basic, Pharmacia) and gel electrophoresis (Bio-Rad). Tetramers were generated by adding phycoerythrin (PE)-labelled streptavidin (Molecular Probes) at a ratio of 1.5:1. We generated HLA-B27 tetramers with the EBV EBNA peptide (residues 258–266) [24]. For the detection of *Chlamydia*-peptide-specific CD8^+ ^T cells we used the previously described immunodominant peptides 8, 68, 80, 131, 133, 138, 144, 145, 146, 194, 195 and 196 [10] (Table 2). Peptides 144 and 194 caused heavy aggregation during refolding procedure and were excluded from tetramer staining; peptide 146 was excluded because of high background staining in more than 50% of the patients.

HLA-A2 monomers with the EBV peptide [21] were generated with an HLA-A2 heavy chain (gift from Dr KH Lee, Berlin, Germany) with the same protocol.

For FACS analysis, frozen mononuclear cells (MNCs) from synovial fluid or peripheral blood were incubated with tetramer and PerCP-labelled anti-human CD8 antibody (BD Pharmingen, San Diego, USA) in parallel for 30 min at room temperature (20°C) followed by washing twice with phosphate-buffered saline (PBS)/2% bovine serum albumin (BSA) and incubation with Cy5-labelled anti-human CD3 antibody for 30 min at room temperature. Cells were washed twice in PBS/2% BSA and resuspended in Annexin V buffer (Molecular Probes) and 2.5 μl of Alexa 488-labelled Annexin V (Molecular Probes) was added. CD8^+ ^and tetramer-positive T cells were analysed after gates were set on CD3^+ ^and Annexin V-negative cells. Depending on the availability of additional synovial lymphocytes we repeated the staining experiments, which was true for the synovial fluid of patient no. 6.

### T cell lines from magnetic activated cell sorting (MACS)-sorted HLA-B27 tetramer-positive CD8^+ ^T cells

Peripheral MNCs were incubated for 30 min with Cy5-labelled anti-CD8 antibody (BD) and 5 μg/ml PE-labelled HLA-B27/EBV EBNA (258–266) tetramer at room temperature. Cells were washed twice and incubated for 15 min at 4°C with anti-PE-labelled MACS beads (Miltenyi) at a ratio of 20 μl of beads to 80 μl of cell suspension. Labelled cells were loaded on an LS MACS column (Miltenyi) and eluted after the column had been washed three times with washing buffer including PBS, EDTA and BSA. MACS-sorted tetramer-positive and CD8^+ ^T cells were further separated by FACS sorting. Sorted cells (1000) were incubated with 500,000 autologous antigen-presenting cells in the presence of 20 U/ml interleukin (IL)-2, 10 ng/ml IL-7 and 10 ng/ml IL-15 added every 3–4 days.

### Determination of the refolding rate of recombinant HLA-B27 monomers

The refolding rate of recombinant HLA-B27 monomer was analysed by gel filtration and by determining the relative amount of soluble HLA-B27 monomer eluted at 13.7 ml in comparison with precipitated protein eluted earlier in a Superose 12 column (Pharmacia). An Akta basic system (Pharmacia) was used. The elution profile was analysed by using Unicorn (version 4) software (Pharmacia). Refolding was defined as ++ when more than 75% of proteins loaded on the gel filtration column after refolding, biotinylation and sharp centrifugation was soluble HLA-B27 monomer molecule; + for more than 50% soluble HLA-B27 monomer, (+) for more than 10% soluble HLA-B27 monomer, and - for less than 10% soluble HLA-B27 monomer (Table 2).

### FACS analysis of intracellular cytokine staining

Intracellular cytokine staining was used after antigen-specific T cell stimulation. Synovial MNCs and peripheral MNCs were stimulated for 6 hours in 1 ml of culture medium with anti-CD28 antibody (1 μg/ml) plus single peptides (10 μg/ml) or without antigenic peptide as a negative control. Brefeldin A was added after 2 hours to stop the stimulation, and cells were harvested after a further 4 hours and then stained with 5 μg/ml anti-CD69-PE antibody (BD Pharmingen) and 1 μg/ml anti-CD8-PerCP (BD Pharmingen). Cells were then fixed in 2% formalin and resuspended in saponin buffer, followed by incubation with 1 μg/ml Cy5-conjugated anti-human interferon-γ antibody (IFN-γ; BD). Gated CD8^+ ^T cells that were positive for early activation marker CD69 and for intracellular IFN-γ were counted as antigen-specific. Analysis was performed with a BD Biosciences FACScan flow cytometer with CellQuest software.

### Infection of peripheral-blood-derived dendritic cells *in vitro *with viable *Chlamydia trachomatis*

CD14^+ ^cells from peripheral blood were incubated for 1 hour with anti-CD14-conjugated magnetic beads (Miltenyi) and sorted by MACS. The purity of separated cells was confirmed by FACS analysis. Cells (500,000) were cultured for 7 days in 24-well plates at 37°C at 5% CO_2 _in 1 ml of RPMI culture medium supplemented with 10% fetal calf serum, 2 mM L-glutamine and 50 ng/ml granulocyte/macrophage colony-stimulating factor and 10 ng/ml IL-4 to induce transformation to dendritic cells (DCs). Cells were washed and harvested and incubated for 24 hours with infectious elementary bodies of *Chlamydia trachomatis *at a ratio of 1:50. DCs were analysed by FACS with the use of anti-CD80, anti-CD86, anti-HLA-DR, anti-CD14 (BD Pharmingen) and anti-*Chlamydia trachomatis *lipopolysaccharide antibodies (Dako) before and after infection with viable *Chlamydia trachomatis*.

### Expansion of *Chlamydia*-specific CD8^+ ^T cells *in vitro *with *Chlamydia*-infected peripheral-blood-derived dendritic cells

We stimulated CD8^+ ^T cells from peripheral blood with *Chlamydia trachomatis*-infected peripheral-blood-derived DCs at a ratio of 50:1 in RPMI culture medium supplemented with 10% fetal calf serum, 2 mM L-glutamine, 100 U/ml penicillin and 100 μg/ml streptomycin. Recombinant IL-7 (10 ng/ml) and IL-15 (10 ng/ml) were added on both days 2 and 7. T cells were analysed by FACS on day 14.

## Results

### MHC class I tetramer staining with HLA-A2/EBV peptide-specific and HLA-B27/EBV peptide-specific tetramers

**Figure 1:**
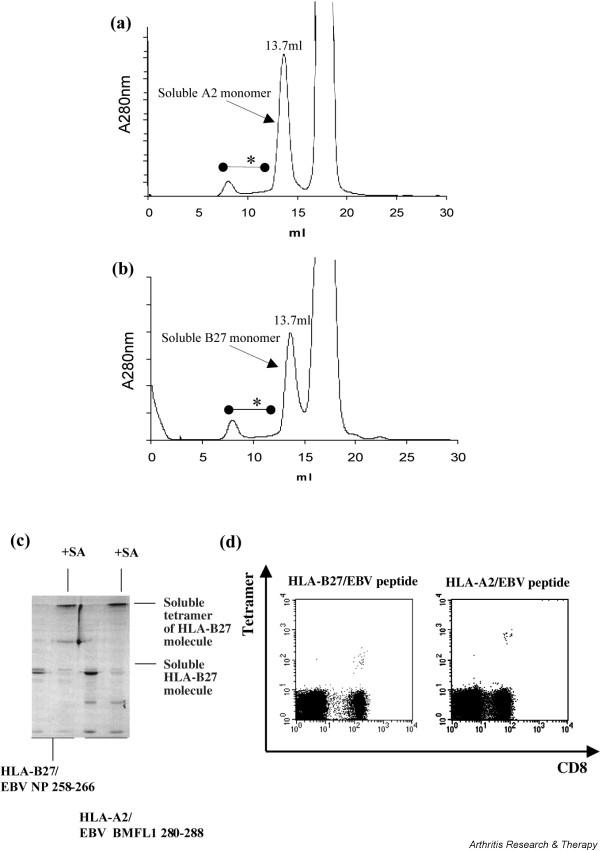
Gel filtration and gel electrophoresis of refolded HLA-A2/EBV peptide monomers **(a**; **c**, lanes 3 and 4) and HLA-B27/EBV peptide monomers **(b**; **c**, lanes 1 and 2). In both experiments the amount of protein aggregation is low, indicated by small amounts of eluted proteins between 7 and 13 ml. The peaks eluted at 13.7 ml contained the soluble MHC monomer. The gel in (c) shows that both eluted monomers are highly purified (lanes 1 and 3) and that most soluble monomers bind to streptavidin if added (lanes 2 and 4). **(d) **Antigen-specific T cells could be detected with both tetramers, although HLA-A2 tetramers stained with greater intensity (log 0.8 more) than HLA-B27. *A*_280_, absorption at 280 nm; asterisks, protein aggregates; SA, streptavidin.

To determine optimal conditions for the refolding procedure of soluble HLA-B27 monomers we used HLA-A2 tetramers and HLA-B27 tetramers with well-described HLA-B27 and HLA-A2 restricted viral epitopes from EBV. The binding scores of the two immunodominant EBV peptides to the HLA-A2 [21] (score 29) and HLA-B27 [24] (score 28) receptor were almost identical in the SYFPEITHI program. By generating the HLA-B27 tetramer (Fig. 1a,1c; lanes 1 and 2) and the HLA-A2 tetramer (Fig. 1b,1c; lanes 3 and 4) the percentages of protein aggregates eluted between 7 and 13 ml in gel filtration, and the refolded monomer eluted at 13.7 ml in gel filtration, were also similar. The large peak at 16 ml most probably contained reagents from the biotinylation reaction because we did not detect any proteins with a molecular mass of more than 5 kDa by SDS-PAGE. This peak was excluded when the relative amount of soluble HLA-B27 monomers was estimated. In FACS analysis, tetramer-positive antigen-specific T cells could be detected with both tetramers (Fig. 1d), although HLA-A2 tetramers stained with greater intensity (log 0.8 more) than the HLA-B27. On the basis of these results we generated HLA-B27/*Chlamydia *peptide tetramers.

### Generation of antigen-specific CD8^+ ^T cell lines after MACS sorting of HLA-B27 tetramer-positive T cells

**Figure 2:**
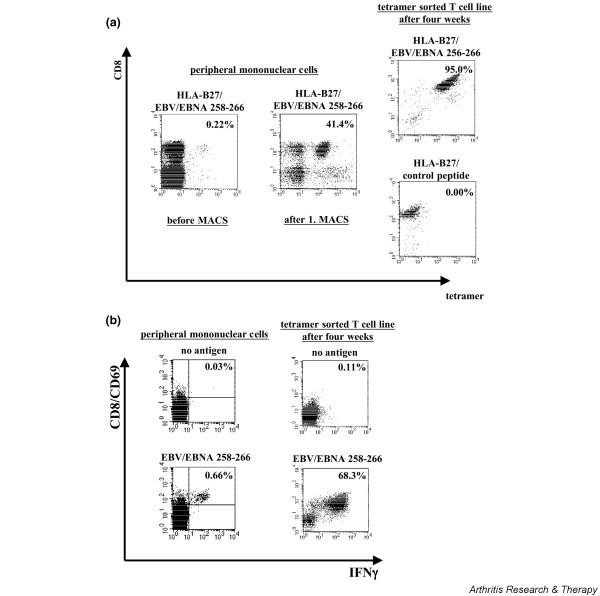
HLA-B27/EBV tetramers and use of magnetic beads to enrich for HLA-B27-restricted tetramer-positive CD8^+ ^T cells. In peripheral blood from an HLA-B27^+ ^blood donor, 0.22% EBV EBNA (258–266) peptide-specific T cells were detected with phycoerythrin (PE)-labelled HLA-B27 tetramer **(a) **and 0.66% of CD8^+ ^T cells by performing intracellular cytokine staining of IFN-γ-secreting cells **(b)**. After magnetic activated cell sorting with anti-PE magnetic beads, antigen-specific T cells were enriched to 41.4% (a). These cells were further purified by fluorescence-activated cell sorting (not shown) and cultured for 4 weeks. After 4 weeks of culturing in the presence of interleukin-2, 95% of the T cell line were antigen-specific as shown by HLA-B27 tetramer staining (a), whereas only 68.3% of antigen-specific T cells could be identified by intracellular cytokine staining (b).

To determine whether tetramer-binding CD8^+ ^T cells could be sorted and further cultured we stained peripheral MNCs with HLA-B27/EBV EBNA (258–266) tetramer. Before MACS, 0.22% of peripheral MNCs were CD8^+ ^and tetramer-positive. After MACS, EBV EBNA (258–266)-specific T cells were enriched to 41.4%. MACS-sorted cells were further separated by FACS sorting and cultured for 4 weeks in the presence of IL-2. The purity of antigen-specific CD8^+ ^was increased to 95.0%, as shown by tetramer staining (Fig. 2a). In parallel we performed intracellular cytokine staining after peptide-specific stimulation of peripheral MNCs and of the tetramer-sorted T cell line after 4 weeks of culture. In comparison with HLA-B27 tetramer staining, only 68.3% of these antigen-specific T cells were detected by intracellular cytokine staining of IFN-γ (Fig. 2b).

### Generating HLA-B27/*Chlamydia *peptide tetramers

**Figure 3:**
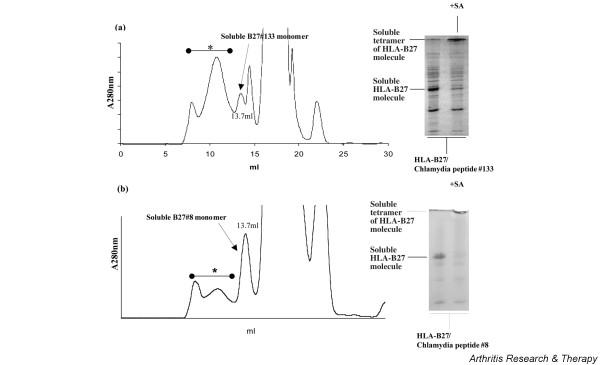
Gel filtration and gel electrophoresis of refolded HLA-B27/*Chlamydia *peptide monomers loaded with *Chlamydia *peptide 133 **(a) **and with *Chlamydia *peptide 8 **(b)**. The peaks eluted at 13.7 ml contained the soluble MHC monomer. The amount of refolded HLA-B27 monomer with *Chlamydia *peptide 8 (b) was higher than that of peptide 133 (a) with less protein aggregation (proteins eluted between 7 and 13 ml), indicating that peptide 8 has a higher binding affinity for HLA-B27. The SDS-PAGE in (a) and (b) shows that both eluted monomers are the major protein in the eluted fraction and that most soluble monomers bind to streptavidin if added. In both experiments the amount of protein aggregation was higher than refolding of HLA-B27 with a viral epitope, indicated by a greater amount of eluted proteins between 7 and 13 ml (Fig. 1). *A*_280_, absorption at 280 nm; asterisks, protein aggregates; SA, streptavidin.

The generation of HLA-B27 tetramers with *Chlamydia*-derived peptides strongly indicated that the yield of refolded and soluble HLA-B27/*Chlamydia *peptide monomers depended on the binding affinity of the peptide for HLA-B27. Gel-filtration analysis showed that *Chlamydia *peptide 133 (Table 2; binding score 25 in [23]) (Fig. 3a) induced significantly more protein aggregation, seen by protein elution between 7 and 13 ml, than *Chlamydia *peptide 8 (Table 2; binding score 26 in [23] but 10,000 in BIMAS) (Fig. 3b). In SDS-PAGE analysis the large quantity of aggregated proteins is also shown by numerous bands of higher molecular mass (Fig. 3a). After the addition of streptavidin, the major band with biotinylated HLA-B27 molecule could be captured to become a tetramer (Fig. 3a,3b; SDS-PAGE). This phenomenon of protein aggregation depending on the affinity between peptide and HLA-B27 could also be observed with the other *Chlamydia*-derived peptides. The refolding rate of all HLA-B27 tetramers used in this manuscript are summarized in Table 2.

### HLA-B27/*Chlamydia *peptide tetramer staining of synovial T cells

On the basis of our recently identified *Chlamydia*-derived immunodominant peptides in Ct-ReA [10] we successfully synthesized nine HLA-B27 *Chlamydia *peptide tetramers and used them to stain MNCs from the synovial fluid of nine patients (six HLA-B27^+^, three HLA-B27^-^) with Ct-ReA. Four of the six HLA-B27^+ ^patients had a specific T cell binding to at least one HLA-B27/*Chlamydia *peptide tetramer.

The results of tetramer staining in all patients are summarized in Table 3; HLA-B27/*Chlamydia *peptide 195 tetramer bound to the synovial T cells of three (patient nos 2, 3 and 5) of these four patients. Two patients (nos 5 and 6) showed a T cell response to *Chlamydia *peptide 133 as detected by tetramer staining, and one (patient no. 3) had a T cell response to *Chlamydia *peptide 68.

**Figure 4:**
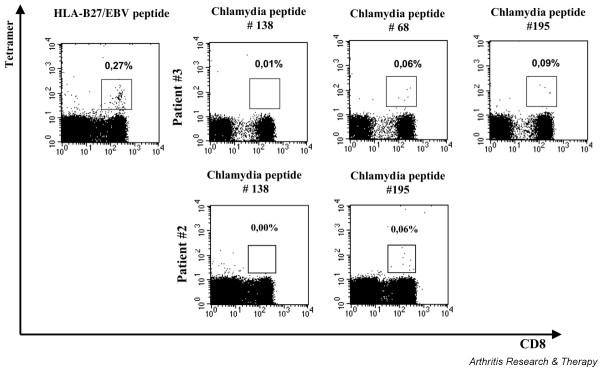
HLA-B27/*Chlamydia *peptide tetramer-binding synovial T cells in patient nos 2 and 3. In patient no. 3, 0.09% *Chlamydia *peptide 195-specific and 0.06% peptide 68-specific CD8^+ ^T cells were identified. All other HLA-B27 tetramers with *Chlamydia*-derived peptides such as peptide 138 did not bind synovial T cells in this patient (data not shown). In patient no. 2, 0.06% *Chlamydia *peptide 195-specific CD8^+ ^T cells were detected by HLA-B27 tetramers. All other HLA-B27 tetramers with *Chlamydia*-derived peptides such as peptide 138 did not bind synovial T cells in this patient (data not shown). Peripheral blood from a blood donor with HLA-B27/EBV EBNA (258–266)-positive CD8^+ ^T cells was used as a control.

**Figure 5:**
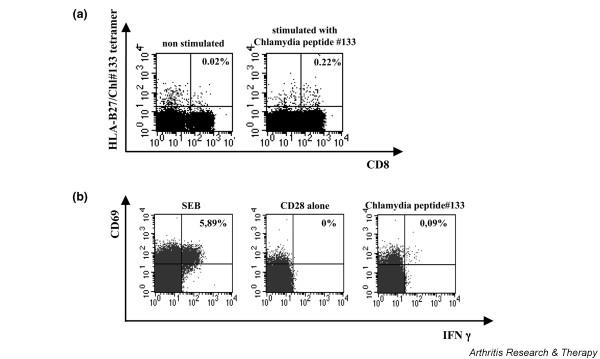
HLA-B27/*Chlamydia *peptide tetramer staining and intracellular cytokine staining in patient no. 6. When synovial T cells where stained with HLA-B27 *Chlamydia *peptide tetramers, only HLA-B27/*Chlamydia *peptide 133 was positive for CD8^+ ^T cells (0.02%). All other HLA-B27 tetramers with *Chlamydia *peptides did not bind synovial T cells in this patient (not shown). The specificity of this staining could be confirmed by an increased quantity of tetramer-positive T cells after peptide-specific T cell stimulation (0.22%) after 1 week of peptide-specific stimulation loaded on synovial mononuclear cells **(a) **and *Chlamydia *peptide 133-specific induction of IFN-γ production in intracellular cytokine staining **(b)**.

The results of three patients are illustrated in Figs 4 and 5. Figure 4a shows that T cells specific for *Chlamydia *peptides 195 and 68 were detected with HLA-B27/*Chlamydia *peptide tetramers in patient no. 3: 0.09% of CD8^+ ^T cells were positive for peptide 195 and 0.06% were positive for peptide 68. All other HLA-B27 tetramers with *Chlamydia*-derived peptides such as peptide 138 were negative (data not shown). In patient no. 2 we detected 0.06% HLA-B27/*Chlamydia *peptide 195 tetramer-positive T cells (Fig. 4b). All other HLA-B27 tetramers such as HLA-B27/*Chlamydia *peptide 138 were negative (data not shown). We did not analyse the cytokine secretion profile of CD8^+ ^T cells in response to chlamydial peptides in these two patients. The example of patient no. 6 is shown in Fig. 5a, with 0.02% of HLA-B27/*Chlamydia *peptide 133 tetramer binding to CD8^+ ^T cells but no binding to any of the other HLA-B27 *Chlamydia *peptide tetramers. In patient no. 6 we were able to repeat this experiment and obtained a similar result, with 0.02% of tetramer binding to CD8^+ ^T cells.

To confirm the specificity of the T cell response to peptide 133, two further experiments were performed in this patient. First, synovial T cells were expanded by *Chlamydia *peptide 133-specific T cell stimulation for 1 week *ex vivo*, which revealed 0.22% tetramer-positive CD8^+ ^T cells (Fig. 5a). Second, when FACS analysis of IFN-γ secretion after peptide-specific stimulation was done in the same patient, only peptide 133 induced this cytokine secretion (Fig. 5b), again confirming the specificity of this response.

We also analysed CD8^+ ^T cells from peripheral blood of patient nos 2, 3, 5 and 6, who were responders when synovial fluid was tested for HLA-B27/*Chlamydia *peptide binding, but we could not detect any specific binding (data not shown). The HLA-B27^- ^patients with Ct-ReA and all six HLA-B27^+ ^healthy controls had no HLA-B27-restricted, *Chlamydia*-peptide-specific T cell response (data not shown). Tetramer staining of synovial T cells from three HLA-B27^+ ^patients with enterobacteria-triggered ReA and from three patients with rheumatoid arthritis revealed no specific staining of CD8^+ ^T cells with 0–0.01% tetramer binding to CD8^+ ^T cells.

### Expansion of *Chlamydia*-specific CD8^+ ^T cells after stimulation with *Chlamydia*-infected dendritic cells

Because the frequency of *Chlamydia*-specific CD8^+ ^T cells in these patients is low in synovial fluid and absent in peripheral blood with both methods (tetramer staining and intracellular cytokine staining), we investigated whether enrichment of these cells could be achieved by short-term stimulation with autologous *Chlamydia*-infected DCs. By doing this we intended to obtain a higher frequency of tetramer-positive CD8^+ ^T cells, to underline the specificity of tetramer staining.

We generated DCs from CD14^+ ^monocytes from peripheral blood of patient no. 5; they were separated by MACS first. After 7 days of cultivation *in vitro*, the cells turned into DCs, as indicated by the loss of CD14 receptors and the upregulation of HLA-DR, CD80 and CD86 receptors (data not shown). We infected these DCs with viable *Chlamydia trachomatis *and confirmed infection by using an anti-*Chlamydia trachomatis *lipopolysaccharide antibody and by quantification of *Chlamydia*-positive cells by FACS analysis (data not shown). We revealed at least 41.3% *Chlamydia*-infected DCs.

**Figure 6:**
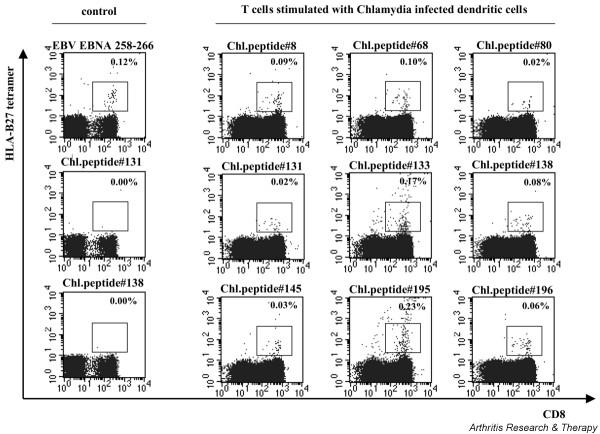
Staining of peripheral blood-derived CD8^+ ^T cell lines generated with *Chlamydia*-infected dendritic cells with nine different HLA-B27/*Chlamydia *peptide tetramers in patient no. 5. A polyclonal T cell response was observed with a significant amount of T cell expansion specific for *Chlamydia *peptides 8, 68, 133, 138, 195 and 196, and there was a weaker response to the other *Chlamydia*-derived peptides.

**Figure 7:**
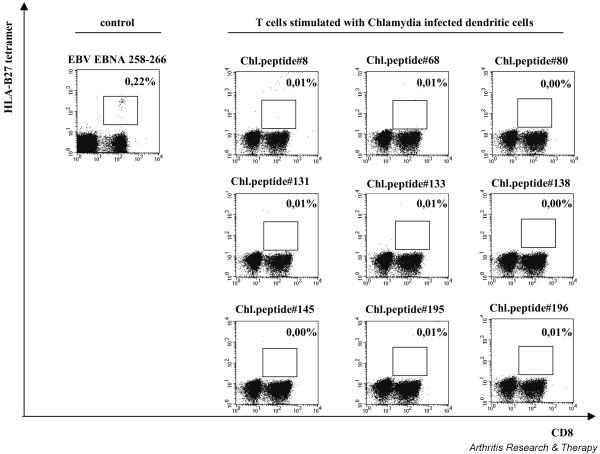
No tetramer staining of peripheral blood-derived CD8^+ ^T cell lines generated with *Chlamydia*-infected dendritic cells with nine different HLA-B27/*Chlamydia *peptide tetramers in the HLA-B27^- ^*Chlamydia*-triggered reactive arthritis (Ct-ReA) patient no. 9. The same experiment was performed in an HLA-B27^- ^patient with Ct-ReA: even after stimulation with autologous *Chlamydia*-infected dendritic cells, *Chlamydia*-peptide-specific CD8^+ ^T cells could not be detected in peripheral blood by HLA-B27/*Chlamydia *peptide tetramers. Chl, *Chlamydia trachomatis*.

Peripheral MNCs from the same patient were stimulated with these *Chlamydia*-infected DCs for 2 weeks in the presence of IL-7 and IL-15. Subsequently, FACS analysis for intracellular cytokine staining for IFN-γ performed after restimulation of this cell line with *Chlamydia*-infected DCs revealed 0.11% IFN-γ-secreting CD8^+ ^T cells, and stimulation with different peptide pools including the nine relevant peptides revealed between 0.07% and 0.21% antigen-specific IFN-γ-secreting CD8^+ ^T cells (data not shown). When the cell line was analysed with HLA-B27/*Chlamydia *peptide tetramers we found a similar quantity of expanded CD8^+ ^T cells with significant tetramer staining of CD8^+ ^T cells specific for *Chlamydia *peptides 8 (0.09%), 68 (0.10%), 133 (0.17%), 138 (0.08%), 195 (0.23%) and 196 (0.06%) (Fig. 6) and a weaker response to the other *Chlamydia*-derived peptides. HLA-B27 tetramer staining with peptides 133 and 195 showed some unusual bright staining, which was also frequently observed with the HLA-B27/EBV EBNA (258–266) tetramer and might have been caused by aggregated tetramers. Staining of untreated peripheral MNCs from the same patient did not reveal any tetramer binding (data not shown); staining with an HLA-B27/EBV peptide tetramer was performed as a positive control. We repeated this procedure in an HLA-B27^- ^patient with Ct-ReA (Fig. 7) and in an HLA-B27^+ ^healthy blood donor (data not shown). In neither case could we observe staining with any of the HLA-B27/*Chlamydia *peptide tetramers even after stimulation with *Chlamydia*-infected DCs (patient no. 9; Fig. 7).

## Discussion

The arthritogenic peptide theory states that some HLA-B27 subtype alleles, owing to their unique amino acid residues, bind one or more specific arthritogenic peptides that are recognized by CD8^+ ^T cells [6-9]. To test this theory it is of great importance to establish methods to identify the peptide specificity of such CD8^+ ^T cells in human beings with HLA-B27-associated arthritis. The use of MHC class I tetramers to detect antigen-specific CD8^+ ^T cells is well established [16]. However, surprisingly few publications present data with HLA-B27 tetramers. We have reported preliminary experiments with HLA-B27 tetramers in single patients with Ct-ReA [10]. HLA-B27 tetramers were also used to determine critical T cell receptor binding regions in HLA-B27-restricted T cells specific for an immunodominant peptide from influenza virus [19]. The biochemical features of the protein might be the limiting factor for using this molecule as frequently as other MHC class I molecules such as HLA-A2 tetramers.

During the refolding process of recombinant HLA-B27, which is expressed in inclusion bodies, significant amounts of aggregated proteins occur [19,20]. The free cysteine residue at position 67 in the HLA-B27 α-chain is chemically highly reactive, causing homodimerization and protein aggregation [19,20,25-27]. It was therefore a reasonable strategy to generate HLA-B27 tetramers by substituting serine for cysteine at position 67 [10,19]. The mutated HLA-B27 heavy chain was also used in these experiments. However, even with the mutated HLA-B27 molecule we experienced significant protein aggregation when HLA-B27 molecules were generated with *Chlamydia*-derived peptides, especially with those with a low binding affinity for HLA-B27. We addressed the question of whether this finding was related to the protocols we used or whether it was specifically related to HLA-B27. We generated an HLA-B27 tetramer with a well-described immunodominant peptide from EBV and compared the results with those for an HLA-A2 molecule also loaded with an immunodominant peptide from EBV having almost the same binding affinity. The refolding rate of both molecules was almost the same, and we obtained comparable results when these molecules were used in FACS analysis. From this we concluded that the use of HLA-B27 tetramers is limited if the binding affinity of a peptide is too low for the molecule to remain stable. We therefore excluded peptides causing heavy protein aggregation and high background staining from further experiments.

Here we have also demonstrated another useful property of HLA-B27 tetramers as a 'proof of principle'. We sorted antigen-specific tetramer-positive CD8^+ ^T cells and generated highly specific T cell lines. After 4 weeks of non-specific stimulation, 95% of T cells were antigen-specific, which could be detected by tetramers but not by intracellular cytokine staining. The latter experiments detected only 68.3% IFN-γ secreting CD8^+ ^T cells after antigen-specific stimulation. These results show clearly that HLA-B27 tetramers have the advantage of detecting antigen-specific T cells independently of their cytokine-secreting profile. Tetramers are also capable of detecting resting antigen-specific T cells, which probably constitute most non-IFN-γ-secreting CD8^+ ^T cells of the T cell line in Fig. 4B (CD69^- ^and IFN-γ^-^).

Using HLA-B27-restricted *Chlamydia *peptides with higher binding scores, previously defined as immunodominant in Ct-ReA [10], we have generated tetramers and identified *Chlamydia*-peptide-specific T cell responses in four of six patients with Ct-ReA. These experiments suggest that *Chlamydia *peptides 195 and 133 are immunologically important epitopes, because we could detect CD8^+ ^T cells with such specificity in three and two, respectively, out of six HLA-B27^+ ^patients. We identified these antigen-specific T cells at a frequency of 0.02–0.09% in the synovial fluid of these patients, which is concordant with previous results [10]. The low frequency of *Chlamydia*-peptide-specific CD8^+ ^T cells detected with HLA-B27 tetramers sometimes makes discrimination from non-specific staining difficult. We confirmed our tetramer staining result in one patient by expansion of peptide-specific CD8^+ ^T cells followed by tetramer staining with increased amounts of tetramer-binding CD8^+ ^T cells; even more importantly, peptide-specific CD8^+ ^T cells were also detected by intracellular IFN-γ staining after peptide-specific stimulation. However, for future experiments it would be useful to confirm such findings with antigen-specific T cell expansion as shown here (Figs 2, 5a and 6a) and also in collaboration with other authors [28].

To underline further the specificity of HLA-B27 tetramer staining we performed peptide-specific expansion of CD8^+ ^T cells specific for *Chlamydia*. For this, we generated *Chlamydia*-infected DCs, which are assumed to be excellent antigen-presenting cells for both CD4^+ ^and CD8^+ ^T cells [29], for *Chlamydia*-antigen-specific CD8^+ ^T cell stimulation; we obtained antigen-specific T cell expansion. This *Chlamydia*-specific CD8^+ ^T cell line showed an increased response to HLA-B27/*Chlamydia *peptides 8, 68, 133, 138, 195 and 196 tetramers and a weaker response to the other HLA-B27/*Chlamydia *peptide tetramers. The generation of CD8^+ ^T cell lines with *Chlamydia*-infected DCs has recently been described, but without defining the MHC restriction and peptide specificity of such T cells [30].

Because we could not detect any *Chlamydia*-peptide-specific CD8^+ ^T cells from peripheral blood with either method (tetramer staining and intracellular cytokine staining) without prior stimulation, we assume that the frequency of CD8^+ ^T cells with such specificity in the peripheral blood is below the sensitivity of both methods. In contrast, low frequencies of *Chlamydia*-derived peptide-specific CD8^+ ^T cells in the peripheral blood were observed by another group by using HLA-A2 tetramers [31]. These researchers detected *Chlamydia trachomatis *major outer membrane protein (MOMP) 258 peptide-specific and MOMP 249 peptide-specific CD8^+ ^T cells in patients with acute urogenital tract infection. They found 0.01–0.2% MOMP-specific CD8^+ ^T cells in the peripheral blood of these individuals with acute infection, who had no clinical symptoms of Ct-ReA.

## Conclusion

We conclude that HLA-B27 tetramers are useful tools for the study of HLA-B27/peptide-specific T cells in HLA-B27-associated diseases. Although *Chlamydia*-specific HLA-B27-restricted CD8 T cells were detected in the synovial fluid of four of six HLA-B27^+ ^patients with *Chlamydia*-induced reactive arthritis, their frequency was low, arguing against a major role in fighting *Chlamydia *and in the pathogenesis of arthritis. However, these antigens might be able to induce a cross-reactive T cell response to self-antigens, implying that the 'arthritogenic peptide' is not necessarily identical with the immunodominant peptide that is capable of inducing the T cell response to eliminate the microbe. Nevertheless, their frequency could be significantly expanded after stimulation *in vitro *with *Chlamydia*-infected autologous DCs, suggesting that these cells have full replicative capacity.

## Competing interests

None declared.

## Abbreviations

BIMAS = BioInformatics and Molecular Analysis Section; BSA = bovine serum albumin; Ct-ReA = *Chlamydia*-triggered reactive arthritis; DC = dendritic cell; EBV = Epstein–Barr virus; FACS = fluorescence-activated cell sorting; IFN-γ = interferon-γ ; IL = interleukin; MACS = magnetic activated cell sorting; MNC = mononuclear cell; MOMP = major outer membrane protein; PBS = phosphate-buffered saline; PE = phycoerythrin.

## Figures and Tables

**Table 1:** Characteristics of patients

Patient no.	B27	Disease	Sex	Age (years)	Disease duration	*Chlamydia *in urine (PCR)	Synovial T cell proliferation	Antibodies
1	+	ReA	Male	18	5 months	+	*Chlamydia trachomatis*	n.d.
2	+	ReA	Male	20	2 months	+	*Chlamydia trachomatis*	n.d.
3	+	ReA	Male	32	5 months	+	*Chlamydia trachomatis*	+
4	+	ReA	Male	34	3 months	+	*Chlamydia trachomatis*	n.d.
5	+	ReA	Male	20	1 month	+	n.d.	+
6	+	ReA	Male	26	1 month	+	*Chlamydia trachomatis*	+
7	-	ReA	Male	56	1 month	+	*Chlamydia trachomatis*	n.d.
8	-	ReA	Male	43	3 months	+	*Chlamydia trachomatis*	n.d.
9	-	ReA	Female	32	6 months	+	*Chlamydia trachomatis*	n.d.
10	+	Chronic ReA	Male	20	7 years	-	Enterobacteria	n.d.
11	+	ReA	Male	47	3 weeks	-	Enterobacteria	n.d.
12	+	ReA	Female	49	6 months	-	Enterobacteria	n.d.
13	n.d.	RA	Female	67	10 years	n.d.	n.d.	n.d.
14	n.d.	RA	Female	49	>1 year	n.d.	n.d.	n.d.
15	n.d.	RA	Female	62	14 years	n.d.	n.d.	n.d.

n.d., not done; PCR, polymerase chain reaction; RA, rheumatoid arthritis; ReA, reactive arthritis.

**Table 2:** Sequence and binding scores of peptides to HLA-B27 and HLA-A2

Name of peptide	Sequence of peptide	Binding score^a^	Binding score^b^	Refolding	Reference
HLA-B27/EBNA (258–266) (EBV)	RRIYDLIEL	28	2000	++	[24]
HLA-A2/BMLF1 lytic antigen peptide 280–288 (EBV)	GLCTLVAML	29	6000	++	[21]
HLA-B27/Influenza NP 383–391	SRYWAIRTR	26	1500	+	[32]
HLA-B27/*Chlamydia *peptide 8	NRFSVAYML	26	10,000	++	[10]
HLA-B27/*Chlamydia *peptide 68	NRAKQVIKL	26	2000	(+)	[10]
HLA-B27/*Chlamydia *peptide 80	IRMFKILPL	26	2000	+	[10]
HLA-B27/*Chlamydia *peptide 131	KRLAETLAL	26	6000	(+)	[10]
HLA-B27/*Chlamydia *peptide 133	IRSSVQNKL	27	2000	(+)	[10]
HLA-B27/*Chlamydia *peptide 138	ARKLLLDNL	26	2000	++	[10]
HLA-B27/*Chlamydia *peptide 144	MRDHTITLL	25	2000	-	[10]
HLA-B27/*Chlamydia *peptide 145	DRLALLANL	27	200	+	[10]
HLA-B27/*Chlamydia *peptide 146	YRLLLTRVL	25	600	(+)	[10]
HLA-B27/*Chlamydia *peptide 194	EREQTLNQL	25	200	-	[10]
HLA-B27/*Chlamydia *peptide 195	NRELIQQEL	25	2000	(+)	[10]
HLA-B27/*Chlamydia *peptide 196	ERFLAQEQL	27	1000	(+)	[10]

Refolding rates are designated as follows: ++, more than 75% soluble HLA-B27 monomer in gel-filtration analysis after refolding and biotinylation; +, more than 50% refolding; (+), more than 10% refolding; -, less than 10% refolding.

^a^SYFPEITHI, HG Rammensee, University of Tübingen [23].

^b^HLA-peptide binding motifs (the BioInformatics and Molecular Analysis Section; ). EBV, Epstein–Barr virus.

**Table 3:** HLA-B27/*Chlamydia *peptide tetramer staining of CD8^+ ^T cells from synovial fluid of six HLA-B27^+ ^patients (nos 1–6) and three HLA-B27^- ^patients (nos 7–9) with *Chlamydia*-triggered reactive arthritis

Patient no.	B27	No. 68	No. 133	No. 195
1	+	-	-	-
2	+	-	-	+
3	+	+	-	+
4	+	-	-	-
5	+	-	+	+
6	+	-	+	-
7	-	-	-	-
8	-	-	-	-
9	-	-	-	-
10	+	-	-	-
11	+	-	-	-
12	+	-	-	-
13	n.d.	-	-	-
14	n.d.	-	-	-
15	n.d.	-	-	-

CD8^+ ^T cells from the synovial fluid of three HLA-B27^+ ^patients with enterobacteria-triggered reactive arthritis (patient nos 10–12), three patients with rheumatoid arthritis (patient nos 13–15) were analysed; the peripheral blood of six healthy HLA-B27^+ ^blood donors was also analysed (not shown). Positive results are indicated with +, no staining with -. All other peptides tested negative. n.d., not done.
